# Supplementary material for: The interactome of intact mitochondria by cross-linking mass spectrometry provides evidence for coexisting respiratory supercomplexes
Source: Mol Cell Proteomics. 2017 Dec 8;17(2):216–32. doi: 10.1074/mcp.RA117.000470 (PMC5795388; doi:10.1074/mcp.RA117.000470)
Supplement: Supplemental Data [file supp_RA117.000470_133922_0_supp_23978_fzffwf.zip › spectra_annotation/mito_DR_spectra_annotation/398-1-1-1-6-1.pdf]

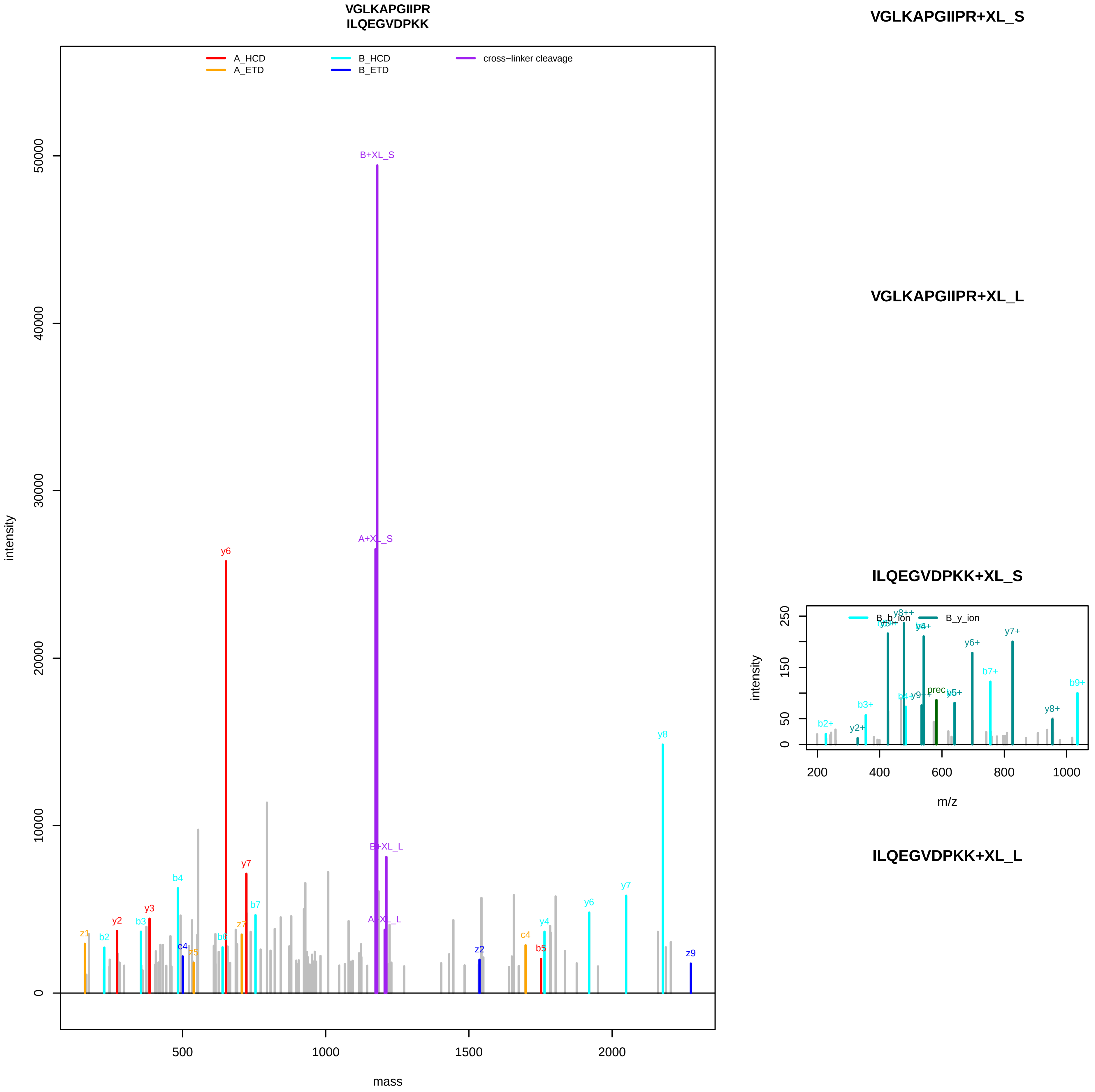
VGLKAPGIIPR
ILQEGVDPKK
A_HCD
A_ETD
B_HCD
B_ETD
cross−linker cleavage
B+XL_S
A+XL_S
B+XL_L
A+XL_L
y6
y8
y7
b4
y3
y2
b3
b2
z1
c4
z5
b6
z7
b7
y4
c4
b5
z2
z9
intensity
mass
0
10000
20000
30000
40000
50000
500
1000
1500
2000
VGLKAPGIIPR+XL_S
VGLKAPGIIPR+XL_L
ILQEGVDPKK+XL_S
B_b_ion
B_y_ion
y8++
y4+
y7+
y6+
b7+
b9+
b3+
b2+
y2+
prec
y5+
y8+
intensity
m/z
0
50
150
250
200
400
600
800
1000
ILQEGVDPKK+XL_L
